# Anchoring Linkage Groups of the Rosa Genetic Map to Physical Chromosomes with Tyramide-FISH and EST-SNP Markers

**DOI:** 10.1371/journal.pone.0095793

**Published:** 2014-04-22

**Authors:** Ilya Kirov, Katrijn Van Laere, Jan De Riek, Ellen De Keyser, Nadine Van Roy, Ludmila Khrustaleva

**Affiliations:** 1 Center of Molecular Biotechnology, Russian State Agrarian University - Moscow Timiryazev Agricultural Academy, Moscow, Russia; 2 Department of Genetics and Biotechnology, Russian State Agrarian University - Moscow Timiryazev Agricultural Academy, Moscow, Russia; 3 Institute for Agricultural and Fisheries Research (ILVO), Plant Sciences Unit, Applied Genetics and Breeding, Melle, Belgium; 4 Center of Medical Genetics, Faculty of Medicine and Health Sciences, Ghent University, Ghent, Belgium; Ecole Normale Superieure, France

## Abstract

In order to anchor *Rosa* linkage groups to physical chromosomes, a combination of the Tyramide-FISH technology and the modern molecular marker system based on High Resolution Melting (HRM) is an efficient approach. Although, Tyramide-FISH is a very promising technique for the visualization of short DNA probes, it is very challenging for plant species with small chromosomes such as *Rosa*. In this study, we successfully applied the Tyramide-FISH technique for *Rosa* and compared different detection systems. An indirect detection system exploiting biotinylated tyramides was shown to be the most suitable technique for reliable signal detection. Three gene fragments with a size of 1100 pb–1700 bp (*Phenylalanine Ammonia Lyase*, *Pyrroline*-*5*-*Carboxylate Synthase* and *Orcinol O*-*Methyl Transferase*) have been physically mapped on chromosomes 7, 4 and 1, respectively, of *Rosa wichurana*. The signal frequency was between 25% and 40%. HRM markers of these 3 gene fragments were used to include the gene fragments on the existing genetic linkage map of *Rosa wichurana*. As a result, three linkage groups could be anchored to their physical chromosomes. The information was used to check for synteny between the *Rosa* chromosomes and *Fragaria*.

## Introduction

Genome structure and function may be studied when comparing the genetic positions of genes with their physical locations on chromosomes. In former times, to assign linkage groups to physical chromosomes it was needed to create monosomic addition lines, nullisomic lines, chromosome substitution lines or translocation lines [1]–[3]. This is a very time consuming task. Nowadays, a more efficient approach exists by direct visualization of genetically mapped markers on chromosomes using fluorescent in situ hybridization (FISH) to locate large genomic clones (BAC, YAC, cosmids etc.) containing the markers. However, FISH with large genomic DNA fragments often results in many non-specific hybridization due to the presence of huge amounts of repetitive DNA in plant genomes [4], [5]. To overcome this problem, FISH using direct labeled individual genes can be applied [6]–[8]. This approach however still is very challenging for most ornamental species and in particular for woody species, such as *Rosa*.

The genus *Rosa*, a member of the Rosaceae, consists of approximately 200 species and 20000 cultivars, most of complex hybrid origin. The genus has a wide phenotypic variability and a high level of genetic heterozygosity [9]. Despite the crop's long domestication history, intensive breeding and economic importance, relatively little is known about the genetics and cytogenetics of roses [10], [11]. Nevertheless, several characteristics of rose make it a worthy candidate for a model system for genomic research in woody species [11].

Performing cytogenetic analyses for roses is difficult because of their genome size (the diploid genome size is 0.83 to 1.30 pg/2C; [12]) and very small chromosomes. The mitotic index is generally low in shoot and root tips, root development is weak and roots are thin in mature individuals for several *Rosa* species [13]. The basic chromosome number of roses is 7 [14], [15] and ploidy levels range from diploid (2n = 2x = 14) to octoploid (2n = 8x = 56) [16]. A number of basic cytogenetic studies, including chromosome counts and karyotyping, have been done on roses [14]–[31]. A karyotype with indication of 45 S and 5 S rDNA sites was constructed for some wild species [24]–[27]. Repetitive sequences, such as 45 S and 5 S rDNA, are rather easy to map, compared to low-copy genes. Reports of physical mapping of low copy genes are found in several genera, such as tomato [32], rice [33], barley [34], wheat [35], sugar beet [36], *Sorghum* [37], maize [7], *Populus trichocarpa* [38] and safflower [39], among others. However, physical mapping of low-copy genes remains a problem in lots of other species and genera and also in *Rosa*. Moreover, in most reports showing conventional FISH results, the target DNA sequences were over 10 kb. Since EST-markers are good candidates to anchor linkage groups to physical chromosomes, lowering the probe-size detection limit should be obtained. Significant improvements in detection limits have been reported, such as the use of a cooled-charge-coupled device (CCD) camera and primed *in situ* DNA labeling (reviewed by Figueroa and Bass [40]). An alternative FISH method used to detect very small probes is tyramide signal amplification (TSA)-FISH, or Tyramide-FISH, a multi-step procedure involving (1) *in situ* hybridization with a labeled probe, (2) signal amplification by streptavidin-horseradish peroxidase (SA-HRP) and tyramides and (3) detection and imaging of the amplified signal [41]. This method was originally introduced by Bobrow et al. [42] for microplate immunoassays. Raap et al. [43] introduced the use of fluorescent tyramide conjugates as substrates for Horse Radish Peroxidase (HRP) into FISH technology. With Tyramide-FISH, the detection sensitivity can be increased up to 100 times compared to the conventional FISH procedures [44]. Tyramide-FISH has been successfully used in human genetics for single-copy gene detection [41], [45]–[52]. In plants, however, Tyramide-FISH has only been used in a few studies [53]–[56].

Molecular markers have been developed in roses to enhance breeding efficiency through the identification and characterization of genes controlling important traits [9], [57], [58]. Major efforts for the construction of genetic linkage maps in the *Rosa* genus have been concentrated at the diploid level [57], [59]–[63,]. Four mapping populations allowed the construction of an integrated consensus map consisting of about 600 markers distributed across 7 linkage groups, with an overall length of 530 cM [58]. Recently, interest in mapping at the tetraploid level has been renewed [64], [65]. Some major rose traits have been located on the rose genetic maps, such as flower color and double corolla [59] and resistance to powdery mildew [61], [62], [63]. To date, no genome sequence is available for the *Rosa* genus that allows validation of the positions of markers located to linkage maps. But *Rosa* is well-supported by the closest sister taxon, which contains the genus *Fragaria*, and also shows sequence homology with *Malus* and *Prunus* [65]–[68]. Developing markers in EST fragments of genes can be based on this sequence homology with other Rosaceae. Although SSRs are widespread in the plant genome, the number of ESTs containing an SSR motif can be quite limited [69]. EST-SNPs have more potential as a functional marker. Due to the conserved nature of the coding sequence, these markers are also appropriate for the comparison of genetic maps between species [70], [71]. High Resolution Melting (HRM) analysis is the method of choice for EST-SNP genotyping, because SNP sequence information is not a prerequisite [72]. HRM was originally introduced as a method for mutation scanning in human genetics [73] and has the ability to simultaneously detect and genotype DNA polymorphisms [74]. The use of HRM for EST-SNP marker development and consecutive mapping in plants has already been reported in several crops such as barley [72], alfalfa [75] and apple [76] but not yet in rose.

The combination of the opportunities of Tyramide-FISH and the HRM molecular marker system may result in an effective integration of physical and genetic maps. The present study had two main aims: 1) to optimize the Tyramide-FISH technology for roses in order to cytogenetically map single-copy genes and 2) to connect their physical position with their genetic position on the linkage groups of *Rosa wichurana* (Moghaddam et al. 2012) using HRM technology.

## Materials and Methods

### Plant Material

The plant material used in this study was *Rosa wichurana, Rosa* ‘Yesterday’ and 90 F1 hybrids of *Rosa* ‘Yesterday’ x *Rosa wichurana*. Both parent plants and the hybrid progeny are diploid (2n = 2x = 14). The plants were own-rooted and grown in the field. For chromosome slide preparations, cuttings of *Rosa wichurana* were made. Rooted cuttings were transferred to terracotta stone pots and grown in the greenhouse without artificial light or temperature regulation. The conditions inside the greenhouse were thus dependent on the moderate climatic conditions typical for the East Flanders region of Belgium.

### Chromosome preparation

Somatic metaphase chromosome spreads were prepared from shoot meristems collected and pretreated according to [13]. Briefly, young shoot meristems (2–3 mm) from which upper green leaves were removed, were collected in ice-cold 1 mM 8-hydroxyquinoline and 0.1% colchicine solution and incubated for 3.5 hours at room temperature in the dark. Afterwards, meristems were fixated in 3∶1 ethanol:glacial acetic acid for 45–60 minutes and stored in 70% ethanol at −20°C. Chromosome slide preparation was carried out according to the spreading protocol of Pijnacker and Ferwerda [77] or to the “SteamDrop” method of Kirov et al. [78].

### Primer and probe design

DNA of *Rosa wichurana*, *Rosa* ‘Yesterday’ and their hybrids was extracted from young leaves using the Qiagen DNeasy Plant Mini Kit (Chatsworth, CA). The genes *PAL*, *P5CS* and *OOMT* were isolated according to Razavi et al. [79] starting from ESTs available in the Genome Database of Rosaceae [80]. These genes are known to be involved in abiotic stress response (*Phenylalanine Ammonia Lyase* (*PAL*) and *Pyrroline*-*5*-*Carboxylate Synthase* (*P5CS*), [81], [82]) and rose scent production (*Orcinol O*-*Methyl Transferase* (*OOMT*), [83]), which are important traits for roses.

To have good probes to use in Tyramide-FISH, we designed primers in order to obtain PCR fragments of about 1500 bp (see Table 1). Plasmid DNA of the cloned gene fragments was labeled using the Biotin Nick Translation Mix (Roche) according to the manufacturer's instructions. As a control, the pTA71 plasmid (containing a 9 kb fragment of 45 S rDNA, [84]) was labeled with biotin.

**Table 1:** Overview of the primers used to isolate the genes PAL (Phenylalanine Ammonia Lyase), OOMT (Orcinol O-Methyl Transferase) and P5CS (Pyrroline-5-Carboxylate Synthase).

Gene	Primers (5′-3′)	Tm (°C)	Source sequence	Amplicon (bp)
*PAL*	ACCACTGGKTTTGGTGCWAC CCYTTGAASCCATAATCCAA	*59.9*	*Prunus persica*	1700
*OOMT*	TGCACTACCAATCCATCCAA TGCCAAGTAACATTTGGCTTT	*59.9*	*Rosa chinensis* ‘*Old Blush*’	1100
*P5CS*	GCTGGCATCCCTGTTGTTAT CTTCGGATCGCTAATGAAGC	59.9	*Prunus persica*	1700

The length of the obtained amplicons is indicated as well as the Tm and source sequence.

To generate EST-SNPs for HRM, we searched for SNPs between *Rosa wichurana* and *Rosa* ‘Yesterday’ in the sequences of the cloned genes *PAL*, *P5CS* and *OOMT*. Primers flanking a single SNP were developed for amplification of the EST-SNPs (Table 2). Primers were tested on the parents and 5 siblings of the mapping population *Rosa* ‘Yesterday’ x *Rosa wichurana*. Good primers were then applied to the entire mapping population.

**Table 2:** Overview of HRM primers for PAL (Phenylalanine Ammonia Lyase), OOMT (Orcinol O-Methyl Transferase) and P5CS (Pyrroline-5-Carboxylate Synthase).

Gene	Primers (5′-3′)	Amplicon (bp)	N° of introns	N° of SNP's
*PAL*	TTGGAGGTTCAAGGAATTTACC CCAAGAAGCGAAAAAGCTCA	227	1	/z
*OOMT*	GTTTGAGGCAGTTCCTCCTG GGTCTTGGTCCAGATCGAGT	223	1	1
*P5CS*	GTGCTTGCAAACATGGAAGA TGGTGCTCTAGTTGGCAAAA	204	1	1

Amplicon length, amount of introns present in the amplicon and the number of SNPs in the amplicon are indicated.

z no sequence information is available for *Rosa wichurana*.

### Tyramide-FISH optimization

Probe hybridization was performed according to Khrustaleva and Kik [53] with minor modifications. Slides were fixed in 4% buffered paraformaldehyde in 1xPBS (10xPBS: 1.3 M NaCl, 70 mM Na2HPO4, 30 mM NaH2PO4, pH 7.5) for 8 min before the RNAse treatment and 10 min before denaturation. Inactivation of endogenous peroxidases was done by incubating the slides in 0.01 M HCl for 8 min. Pepsin treatment was performed during 30 sec at room temperature. The hybridization mixture contained 50% (v/v) deionized formamide, 10% (w/v) dextran sulphate, 2xSSC, 0.25% sodium dodecyl sulphate and 2.00 ng/µl probe DNA. The hybridization mix was denatured at 80°C for 5 min, subsequently placed on ice for 5 min, and added to the chromosome slides. Slides were then denatured for 5 min at 80°C and hybridization was carried out at 37°C overnight. A 82% stringency washing was attained by washing the slides twice in 2xSSC for 5 min at 37°C, twice in 25% (v/v) formamide in 0.4xSSC for 10 min at 42°C, and finally in 2xSSC for 3 min at 37°C.

For probe detection, three tyramide amplification systems were used: direct detection (modified from Schriml et al. [47] and Khrustaleva and Kik [53]), indirect detection (modified from Schriml et al. [47] and Perez et al. [32]) and indirect detection with two rounds of amplification. The incubation time with the tyramide solution vary from 5 to 10 min. In the direct detection system, tyramide-FITC (Tyr-FITC) or tyramide-Cy3 (Tyr-Cy3) was used in dilutions 1∶50. In the indirect detection system, biotinylated tyramides (Tyr-Bio, PerkinElmer, Belgium) were used in the dilutions 1∶25 and 1∶50 and the antibodies (Strepatavidin-Cy3, or Streptavidin-Cy3) were 1∶100 and 1∶300 diluted. The concentration of Tyr-Bio and Streptavidin-HRP (SA-HRP) antibodies used in the first round of the indirect detection with two rounds of amplification system were the same as in the indirect detection system. In the second round of amplification SA-HRP was diluted 1∶300 or 1∶200 and Tyr-Cy3 was used in dilutions 1∶100, 1∶300, 1∶500 or 1∶1000.

Images were taken using a fluorescence microscope Zeiss AxioImager M2 (400x and 1000x magnification) equipped with an AxioCam MRm camera and using Zen software (Zeiss, Zaventem, Belgium). Calculation of chromosome size, centromere index and signal positions was performed using the freeware computer application Micromeasure software, version 3.3 [85].

### Karyotype Analysis

A karyotype was constructed after measurement of five well-spread metaphases using Micromeasure version 3.3 (http://rydberg.biology.colostate.edu/Micromeasure) [85]. Measurements were performed on DAPI stained images and chromosomes were characterized on the basis of chromosome length and centromeric index [86]. Chromosomes were then arranged in order of decreasing length. The condensation index [(genome size 1C (Mbp)/mean length of total chromosome complement (µm)] was also calculated. The FISH signal position (RD) was calculated according to the formula: RD =  distance from signal to centromere ×100%/length of the chromosome arm.

### Genotyping and linkage mapping of EST-SNP markers

HRM was performed as described in [87] but using only the 0.8× LightCycler 480 High Resolution Melting Master Mix (Roche). LightCycler 480 Gene Scanning software was used for genotyping. Three EST-SNPs for the candidate genes *PAL*, *OOMT* and *P5CS* were amplified in the mapping population. A scoring matrix was calculated in Microsoft Excel. Segregation patterns of the new marker sets based on the HRM profiles for the offspring plants of the mapping population were added to the already existing mapping data described in Moghaddam et al. [63]. Estimation of the linkage groups and regression mapping was performed as described in De Keyser et al. [88] using JoinMap 4.0 [89]. Calculation settings for the mapping were: using linkages with a recombination frequency smaller than 0.49 and LOD higher than 1; goodness-of-fit jump threshold for removal of loci 5 and performing a ripple after adding 1 locus. Markers with severe segregation distortion (Chi-square test significance higher than 0.005) and markers creating “tension” in the maps (according to the Nearest Neighbours Fit) were removed from the final maps.

### Determination of the position of OOMT, PAL and P5CS genes on *Fragaria vesca* pseudo-chromosomes

Positions of the *PAL* and *P5CS* genes on the pseudochromosomes of *Fragaria vesca* (FraVesHawaii_1.0) were determined in the gene database at NCBI. Localization of the *OOMT* gene was identified by an alignment of a *Rosa chinensis OOMT1* partial gene sequence (AJ786302) with each of the *F. vesca* pseudochromosome (CM001053.1-CM001059.1) using the BLASTN tool [90]. The E-value threshold was fixed at e-15. To identify the closest strawberry orthologous to the *Rosa wichurana* genes used in our Tyramide-FISH experiments, a BLASTN search against distinct copies of the strawberry genes was performed. As a query, the parts of the *Rosa wichurana* sequences of the *OOMT*, *PAL* and *P5CS* genes corresponding to the gene fragments used in the Tyramide-FISH were used.

## Results

### Tyramide-FISH optimization

**Figure 1:**
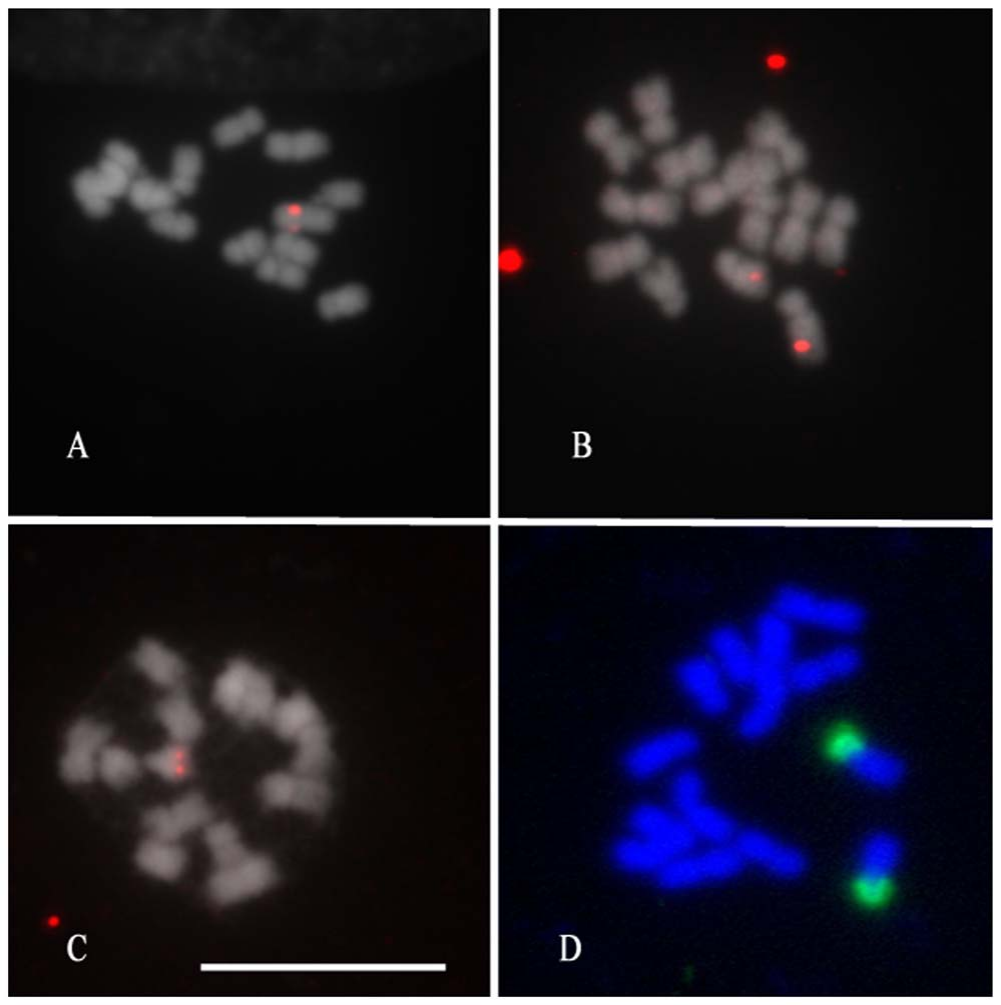
Tyramide-FISH with indirect detection (A, B and C) and direct detection (D) systems on metaphase chromosomes of *Rosa wichurana*. Chromosomes were hybridized with *OOMT* (A), *P5CS* (B), *PAL* (C) and pTA71 plasmid (D). (Bar - 10 µm).

Using the direct detection system to detect the single-copy gene *PAL*, many nonspecific signals were observed, although for the control probe pTa71, 45 S rDNA sites could be detected (Fig. 1D). Therefore, the indirect detection and indirect detection with two rounds of amplification systems were optimized for single-copy gene detection. In the indirect detection system, *PAL* (1700 bp) could be observed when using a 1∶25 dilution rate for Tyr-Bio, 8–10 minutes tyramide incubation time and a 1∶100 dilution rate for SA-Cy3. These conditions gave the best signal-to-noise ratio as determined by visual inspection. In the indirect detection with two rounds of amplification system, signals for *PAL* became visible under the following conditions: a first round using SA-HRP (1∶100), Tyr-Bio (1∶25), 5 min tyramide incubation time and a second round using SA-HRP (1∶300), Tyr-Cy3 (1∶500), 6 min tyramide incubation time. Changing the concentration of SA-HRP (1∶200, 1∶300) and Tyr-Cy3 (1∶100, 1∶300, 1∶500 or 1∶1000) in the second round of amplification in the indirect detection with two rounds of amplification system, resulted in slight differences in the signal-to-noise ratio. The optimized indirect detection and indirect detection with two rounds of amplification systems both allowed visualization of the *PAL* signals in 30–40% of the observed metaphases. Because indirect detection is more time consuming than indirect detection, we used indirect detection for the subsequent physical mapping of the genes.

### Physical mapping of genes using ID

**Figure 2:**
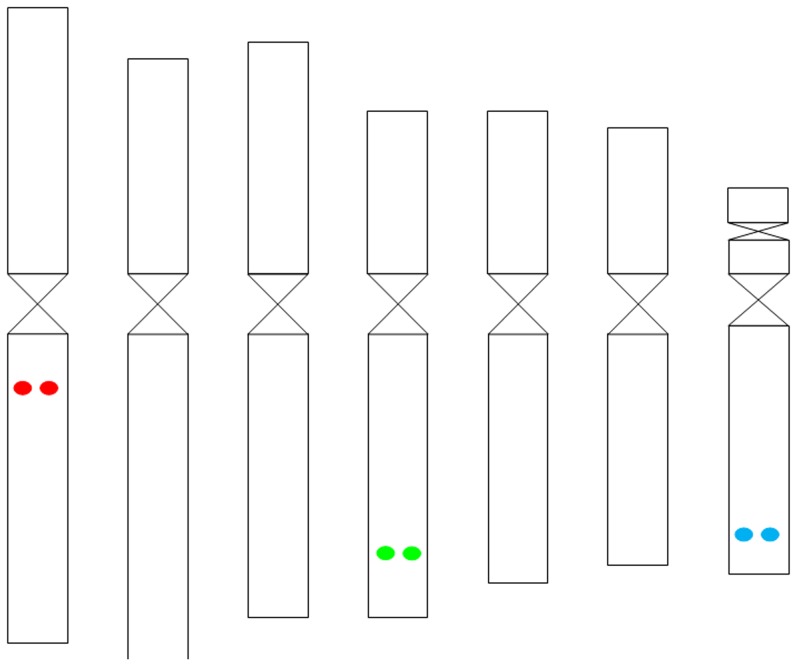
Ideogram of *Rosa wichurana* chromosomes with an indication of the physical position of the candidate genes for *OOMT* (red), *PAL* (blue) and *P5CS* (green).

To be able to link the Tyramide-FISH signals to a certain chromosome and to identify the NOR-bearing chromosome, the karyotype of *Rosa wichurana* was constructed for the first time (Table 3; Fig. 2). The karyotype contains 7 pairs of chromosomes with the chromosome formula 5M+1SM+1ST. The length of the chromosomes ranges between 2.2 µm and 3.7 µm (Table 3). The smallest chromosome bears a NOR-satellite, as confirmed by Tyramide-FISH with 45 S rDNA (Fig. 1 D). Chromosomes 1 and 7 can be easily distinguished based on their size and centromeric indexes. In addition, it is also possible to discern the only submetacentric chromosome 4. The condensation index of *Rosa wichurana* is 28.1±2 Mbp µm^−1^, based on the genome size of *Rosa wichurana* (1C = 562 Mbp; [91]) and the mean total length of the metaphase chromosomes (1n = 20±1 µm).

**Table 3:** Size and centromere index of the *Rosa wichurana* chromosomes.

Chromosome number	Chromosome Length (µm)	Relative Length (%)	Centromere Index (%)
1	3.70±0.30	17.80±0.20	46.00±1.20
2	3.20 0.60	17.00±0.20	40.30±1.30
3	3.00±0.50	15.20±0.20	44.30±1.00
4	2.80±0.40	14.00±0.10	36.90±0.70
5	2.60±0.40	13.60±0.10	41.40±0.70
6	2.50±0.40	12.40±0.20	41.80±1.10
7	2.20±0.50	10.00±0.10	23.40±0.90

The three genes used in this study were mapped on different chromosomes (Fig. 1; Fig. 2). Signals from hybridization of the *PAL* gene were visualized in the distal part of the long arm of the smallest *Rosa wichurana* chromosome 7 (Fig. 1C, Fig. 2) (RD = 77.0±2.1%). The signals were detected in 25–30% of the analyzed metaphases. The *OOMT* gene was visualized in the proximal position of the long arm of chromosome 1 (Fig. 1A, Fig. 2) (RD = 22.6±3.2%). The signals were observed in 30–35% of the analyzed metaphase cells. Tyramide-FISH for the *P5CS* gene resulted in signals on chromosome 4 in 30–40% of the analyzed chromosome spreads. The signals were localized in the distal position (RD = 72.7±3.8%) on the long arm of this chromosome (Fig. 1B, Fig. 2).

### Positioning of EST-SNP on the genetic linkage map

**Figure 3:**
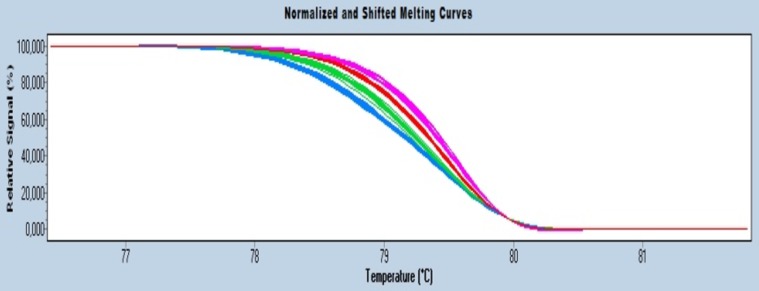
HRM melting profiles for *P5CS*. The melting curve for *Rosa wichurana* is part of the green cluster; *Rosa* ‘Yesterday’ is part of the red cluster. Both clusters also contain curves of the siblings. Blue and pink clusters contain only the melting curves of siblings.

**Figure 4:**
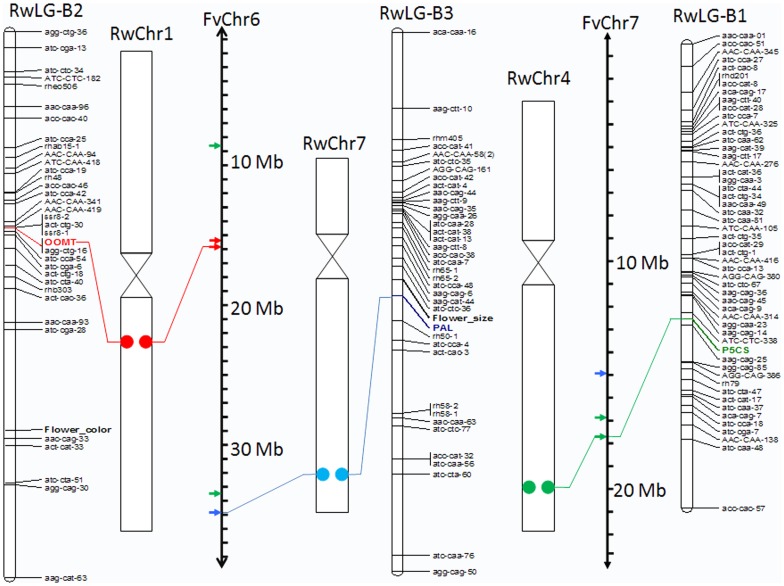
Integration of the gene position on the genetic map (RwLG) (partially) obtained by regression mapping in Joinmap 4.0 showing the consensus linkage groups with indication of the map position of *P5CS* (green), *OOMT* (red), *PAL* (blue) and the physical chromosomes of *Rosa wichurana* (RwChr) and the pseudochromosomes of *Fragaria vesca* (FvChr). Framework of the genetic linkage map follows Moghaddam et al. (2012).

HRM profiles of *OOMT* and *PAL* yielded different melting curves between the parents; melting curves of the offspring were identical to either one of both parental curves. Both markers were scored as <lmxll> according to JoinMap 4.0 [89]. The segregation for *PAL* was slightly distorted (p = 0.005); 64% of the offspring plants were scored as <lm>. For *OOMT*, no segregation distortion was detected. The HRM profiles of *P5CS* also differed between the parents and segregated as 4 profiles in the offspring plants (2 of them were identical to the parental profiles; Fig. 3). Hence, this marker was scored co-dominantly as <efxeg> according to JoinMap 4.0 [89] in a ratio of 23∶19∶19∶29 for ee:ef:eg:fg, respectively. No segregation distortion was present for *P5CS*. Segregation pattern-derived EST-SNP markers for *PAL*, *OOMT* and *P5CS* were integrated in the existing genetic linkage maps of Moghaddam et al. [63] (Fig. 4). *P5CS* was inserted into consensus linkage group RwLG-B1; *OOMT* in group RwLG-B2 and *PAL* into group RwLG-B3 (Fig. 4). The OOMT gene was previously mapped on linkage group 2 [58], [105] that correspond to our RwLG-B2. Two morphological traits, “flower size” (*Rosa* ‘Yesterday’ has double flowers, *Rosa wichurana* has simple flowers) and “flower color” (*Rosa* ‘Yesterday’ has pink flowers, *Rosa wichurana* has white flowers), were recorded as qualitative traits in the mapping population [63]. “Flower size” and “flower color” are very old and well-known loci in rose linkage maps. The traits were scored in the mapping population *Rosa* ‘Yesterday’ x *Rosa wichurana* during 3 years in a qualitative manner [63]. A close linkage between *PAL* and “Flower size” (3 cM) was observed. *OOMT* and “Flower color” are on the same linkage group but with a larger linkage distance (36 cM). Genetic mapping and Tyramide-FISH results are in concordance as the three genes were mapped on three different chromosomes and linkage groups. The position of the *PAL* and *P5CS* genes near the end of the linkage groups correspond with their positions on the chromosomes, which is also relative to the telomeric ends (Fig. 4). The relative position of *OOMT* is central on RwLG-B1 and has a proximal position on chromosome 1 (Fig. 4).

### Anchoring of linkage groups to *Rosa wichurana* chromosomes and *Fragaria vesca* pseudochromosomes

Searching for orthologous genes for *OOMT*, *P5CS* and *PAL* genes in strawberry genome revealed that they are represented in 3, 4 and 2 genes paralogous, respectively (Table 4). Sequence alignment showed that sequence diversity between the paralogous ranges from 66% (for *OOMT*) to 91% (for *P5CS*) (Table 4). Two paralogous *OOMT* genes are located on strawberry pseudochromosome 6 (FvChr6) and one on FvChr3. Two paralogous *P5CS* genes are located close to each other on FvChr7 and two on FvChr6. Paralogous for the *PAL* genes were found on FvChr6 and FvChr7. BLASTN comparison between the sequences of OOMT, P5CS and *PAL* from *Rosa wichurana* and all found paralogous in strawberry, revealed that three strawberry paralogues (highlighted in Table 4) show a high similarity and/or sequence coverage to the rose genes. These paralogues are used for making a comparison between the physical locations of *OOMT*, *P5CS* and *PAL* genes on the strawberry pseudochromosomes and the *Rosa wichurana* chromosomes (Fig. 4). *OOMT* is located in the centre of FvChr6 (Fig 4) and, as revealed in our Tyramide-FISH, in the centromeric region on chromosome 1 of *Rosa wichurana* (RwChr1; Fig 4). *PAL* is located distally on FvChr6 (Fig 4) and distally on chromosome 7 of *Rosa wichurana* (RwChr7; Fig 4)). *P5CS* is located distally on pseudochromosome FvChr7 (Fig 4) and on the distal part of *Rosa wichurana* chromosome 4 (RwChr4; Fig 4).

**Table 4:** Divergence among members of *PAL*, *P5CS*, *OOMT* orthologous genes of *Fragaria* and their similarity to *Rosa wichurana* gene fragments used in this study.

Gene	Number of orthologous genes found in *Fragaria*	*Fragaria* orthologous gene localizations	Similarity between *Fragaria* orthologous genes	% similarity to *Rosa wichurana* gene fragments (E-value; % coverage)
PAL	2	FvChr7:15014006–15017322 FvChr6:34874086–34877587	76%	75% (3e-35; 20%) 83% (0.0; 65%)
P5CS	4	FvChr7: 17624431–17630820 16924786–16929803 FvChr6: 8598452–8605103 33424492–33427031	78%–91%	82% (0.0;99%) 88% (2e-52;37%) 79% (2e-15; 21%) Not significant^z^
OOMT	3	FvChr3: 7085125–7086298 FvChr6: 15275992–15277245 15267146–15267850	66–67%	70% (8e-37; 87%) 91% (0.0; 88%) 44% (8e-47; 62%)

Genes that were selected for the comparative analysis are highlighted.

z : Not significant: according to BLAST search.

## Discussion

### Short DNA fragments could be visualized on physical chromosomes using Tyramide-FISH

To the best of our knowledge this study reports the first successful use of Tyramide-FISH in a plant genus with small chromosomes. Previously, Tyramide-FISH has been applied to visualize short DNA fragments for large chromosomes of several monocots including onion [53], [92], barley [54], wheat [55] and oat [56]. Despite the difficulty of using rose as a cytogenetic object, we successfully visualized short DNA fragments (1.1–1.7 Kb) of genes using Tyramide-FISH. Although rose chromosomes are very small, the degree of chromosome condensation is rather low (28.1±2 Mbp µm^−1^). This value is comparable with tomato (40.6 Mbp µm^−1^, [93]) and humans (26.6 Mbp µm^−1^, [94]), but is more than seven times lower than in onion (249.6 Mbp µm^−1^, [95]). The nature of chromosome structure and chromatin compaction influences the accessibility of target DNA. Low chromatin compaction may positively influence the Tyramide-FISH sensitivity by improving the probe penetration into the chromosomes. On the other hand, less compact chromatin theoretically can have a negative impact on Tyramide-FISH because it contains smaller amounts of proteins (e.g., histones) and electron rich amino acids (e.g., tyrosine, tryptophan) around the site of hybridization. Tyramides, used for signal amplification, are phenolic compounds that react and bind with these electron rich moieties in the presence of HRP and hydrogen peroxide. Therefore, a smaller amount of electron rich amino acids can hamper a successful tyramide-conjugate coupling reaction after oxidation by HRP [42].

We found that the commonly-used direct detection system with fluorescent labeled tyramides (Tyr-FITC, Tyr-Cy3) was not suitable for rose chromosomes. In that system, many nonspecific signals hampered the identification of signals from the *PAL* gene. Optimization using the indirect detection and indirect detection with two rounds of amplification overcame this problem. The indirect detection system has previously been applied to detect the *Rad51* gene on wheat chromosomes [55] and several EST clones on human chromosomes [47]. In the study of Schriml et al. [47], the indirect detection system using avidin-FITC provided the best results, i.e., clear, distinct signals on one or both of the homologues; whereas both the Tyr-Cy3 and Tyr-FITC (direct detection) resulted in high background [47]. The frequency of signal detections was about 30–40% in our study. This is comparable with previous studies. In the study of Perez et al. [55], the Tyramide-FISH procedure using Tyr-Bio was able to detect target DNA sequences as small as 2 kb with a frequency of 37.5%. These frequencies are high enough to unequivocally locate small sequences (<2 kb) using a few metaphase cells and shows the effectiveness of our Tyramide-FISH detection system. In most cases, we observed the Tyramide-FISH signals only on one homologous. The same results were obtained on wheat [55] and *Allium* (Kirov et al. unpublished data) where short DNA probes were used. Since chromatin structure significantly influences FISH results, the unequal distribution of the signals among the homologous and the low frequency of the signals may be the results of variation in chromatin accessibility and/or chromatin disorder between chromosomes and metaphase plates, caused by chromosome preparation procedure.

### The HRM technology for EST-SNP marker generation has several advantages

We successfully visualized the position of the *OOMT*, *P5CS* and *PAL* genes on the *Rosa wichurana* chromosomes 1, 4 and 7, respectively. Using EST-SNP markers for these genes, we could anchor three linkage groups of *Rosa wichurana* to their physical chromosomes for the first time. EST-SNP markers made it possible to connect the physical position of the *OOMT*, *P5CS* and *PAL* genes with their position on the genetic map. The HRM technology allowed detecting SNPs in a fast and efficient way. Unlike other technologies for gene mapping, HRM can be applied immediately after PCR without further handling [73]. During a single two-hour assay we amplified all 3 genes in a single-step procedure on a 384-well plate. This dramatically increases the genotyping throughput in a mapping population. Curve shapes cannot always be assigned to specific alleles [96], but this was not the case here. EST-SNP markers are situated in functional genes, therefore these markers are a valuable tool for the integration of the physical and genetic position of genes.

### Tyramide-FISH showed single loci for members of multigene families

Surprisingly, by using Tyramide-FISH we only observed single loci for each gene even though they were described as members of multigene families [97], [98]. To estimate the copy number of genes in a plant genome, a collection of EST sequences can be used [99]. For roses, more than 20000 rose EST sequences were uploaded in NCBI [83], [100]–[102] of which only 1936 EST sequences [101] belong to *Rosa wichurana*. This number of EST sequences is not enough for the estimation of the copy number of the three genes that we studied *in Rosa wichurana* even not if EST sequences from another *Rosa* species would be used in our analysis. Variations in EST sequences can be explained by the copy numbers of a gene but also by allelic variations. Some *Rosa* species may have up to 16 allelic variants (for ploidy level 2n = 8x) per gene. Therefore, for a correct estimation of the copy number of the genes in *Rosa wichurana* using a database of EST sequences, it should contain more sequences (e.g. 120892 ESTs were used for tomato [99]) of cDNA clones isolated from different tissues. Moreover, an EST library represents only expressed genes and does not include pseudogenes that can be visualized by Tyramide-FISH.

To clarify our result we performed BLASTN searches of all *PAL*, *OOMT* and *P5CS* genes known in *Fragaria,* the closest relative of *Rosa* [65], [103]. It has a completely sequenced genome [104]. We found 2, 4 and 3 hits for the *PAL*, *P5CS* and *OOMT* genes, respectively, distributed along 3 *Fragaria* pseudochromosomes 3, 6 and 7. However, the similarity between the *Fragaria* orthologous genes (66–76%) for *OOMT* and *PAL* genes is low. The 4 *Fragaria* orthologous genes for *P5CS* genes showed a higher level of intragenic similarity, but a pairwise alignment with the rose gene fragment for *P5CS* used in our Tyramide-FISH indicated only one strawberry orthologous gene with a high similarity (82%) and query coverage (99%). Therefore, if the rose genome contains a similar copy number of *PAL*, *OOMT* and *P5CS* and with similar intragenic differences as in the *Fragaria vesca* genome, with the hybridization and washing stringency we used in our study, we can specifically detect the particular orthologues PAL, OOMT and P5CS genes with high homology to the probe DNA sequence. Thus, for each orthologue we can get a clear locus on the chromosomes, which is a very important feature for anchoring linkage groups to physical chromosomes.

### Comparative analysis of physical gene positions between *Rosa wichurana* and *Fragaria vesca*

A comparison of the physical position of the three genes between the *Rosa wichurana* chromosomes and the *Fragaria vesca* pseudochromosomes revealed that FwChr6 contains both orthologous *PAL* and *OOMT* genes, although they are located on different chromosomes of *Rosa wichurana*. Previously, Gar et al. [65] genetically mapped a set of orthologous EST markers on *Rosa* and compared this with their position on the *Fragaria vesca* chromosomes. They showed 10 rearrangements including 4 translocations and 6 inversions changing the gene order between *Rosa* and *Fragaria vesca* chromosomes. One of these rearrangements involved FwChr6, which was shared by markers from 2 *Rosa* linkage groups. Our results are thus in accordance with Gar et al. [65]. Physical mapping on the rose chromosomes of additional genes present on FwChr6 will shed light on the nature and the scale of this rearrangement.

In conclusion, our results demonstrate that Tyramide-FISH is a useful tool for physical mapping of short DNA fragments of genes on *Rosa* chromosomes. We could physically map 3 genes on the chromosomes of *Rosa wichurana*. Using the opportunities of the Tyramide-FISH and the HRM technology, 3 linkage groups could be anchored to 3 physical chromosomes of *Rosa wichurana*. An integration of a cytogenetic and genetic map of rose is an indispensable tool for assistance in map based cloning. Moreover, the information obtained from the physical mapping of individual rose genes can be applied for contig and pseudochromosome anchoring to physical chromosomes which will assist future genome sequencing in *Rosa*.
